# Association of Undifferentiated Dyspnea in Late Life With Cardiovascular and Noncardiovascular Dysfunction: A Cross-sectional Analysis From the ARIC Study

**DOI:** 10.1001/jamanetworkopen.2019.5321

**Published:** 2019-06-14

**Authors:** Sergio H. R. Ramalho, Mario Santos, Brian Claggett, Kunihiro Matsushita, Dalane W. Kitzman, Laura Loehr, Scott D. Solomon, Hicham Skali, Amil M. Shah

**Affiliations:** 1 Health Sciences and Technologies Post-Graduation Program, University of Brasília, Brasília, Brazil; 2 Division of Cardiovascular Medicine, Brigham and Women’s Hospital, Boston, Massachusetts; 3 Department of Physiology and Cardiothoracic Surgery, Cardiovascular R&D Unit, Faculty of Medicine, University of Porto, Porto, Portugal; 4 Department of Epidemiology, Johns Hopkins Bloomberg School of Public Health, Baltimore, Maryland; 5 Wake Forest School of Medicine, Winston-Salem, North Carolina; 6 Gillings School of Public Health, University of North Carolina at Chapel Hill, Chapel Hill

## Abstract

**Importance:**

Undifferentiated dyspnea is common in late life, but the relative contribution of subclinical cardiac dysfunction is unknown. Impairments in cardiac structure and function may be characteristics of undifferentiated dyspnea in elderly people, providing potential insights into occult heart failure (HF).

**Objective:**

To quantify the association of undifferentiated dyspnea with cardiac dysfunction after accounting for other potential contributors.

**Design, Setting, and Participants:**

This cross-sectional study used data from Atherosclerosis Risk in Communities study participants 65 years and older who attended the fifth study visit (from 2011 to 2013) and had not been diagnosed with HF, chronic obstructive pulmonary disease, morbid obesity, or severe kidney disease. Analyses were conducted from October 2017 to June 2018.

**Exposures:**

Dyspnea measured using the modified Medical Research Council scale, with a score less than 2 classified as none to mild and a score of 2 or more classified as moderate to severe.

**Main Outcomes and Measures:**

Using multivariable logistic regression, the association of undifferentiated dyspnea was defined using cardiac structure, systolic and diastolic function, pulmonary pressure (echocardiography), pulmonary function (spirometry), glomerular filtration rate, hemoglobin, body mass index, depression, and physical performance. The population-attributable risk was calculated for each dysfunction metric.

**Results:**

Among 4342 participants (mean [SD] age, 75.9 [5.0] years; 2533 [58.3%] women), 1173 (27.0%) had undifferentiated dyspnea. Moderate to severe dyspnea was present in 574 participants (13.2%) and was associated with left ventricular (LV) hypertrophy (odds ratio [OR], 1.53; 95% CI, 1.25-1.87; *P* < .001) and LV diastolic (OR, 1.46; 95% CI, 1.20-1.78; *P* < .001) and systolic (OR, 1.28; 95% CI, 1.05-1.56; *P* = .02) dysfunction. Moderate to severe dyspnea was also associated with obstructive (OR, 1.59; 95% CI, 1.28-1.99; *P* < .001) and restrictive (OR, 2.56; 95% CI, 1.99-3.27; *P* < .001) findings on spirometry, renal impairment (OR, 1.32; 95% CI, 1.08-1.61; *P* = .01), anemia (OR, 1.72; 95% CI, 1.39-2.12; *P* < .001), lower (OR, 2.77; 95% CI, 2.18-3.51; *P* < .001) and upper (OR, 1.82; 95% CI, 1.49-2.23; *P* < .001) extremity weakness, depression (OR, 3.01; 95% CI, 2.24-4.25; *P* < .001), and obesity (OR, 2.35; 95% CI, 1.95-2.83; *P* < .001). After accounting for these, moderate to severe dyspnea was associated with LV hypertrophy (OR, 1.30; 95% CI, 1.01-1.67; *P* = .04) and was not associated with systolic or diastolic function. In contrast, in the fully adjusted model, other organ system measures were associated with dyspnea, except for glomerular filtration rate and grip strength. The population-attributable risk of dyspnea associated with obesity alone was 22.6% compared with 5.8% for LV hypertrophy.

**Conclusions and Relevance:**

Undifferentiated dyspnea is multifactorial in older adults, and this study showed an association with obesity. When accounting for other relevant organ systems, cardiovascular function poorly discriminated those with vs those without dyspnea. Therefore, dyspnea should not be assumed to represent occult HF in this population.

## Key Points

**Question:**

To what extent is undifferentiated dyspnea in late life associated with cardiac dysfunction after accounting for other potentially contributing dysfunctional organ systems?

**Findings:**

In this cross-sectional study of 4342 participants 65 years and older, undifferentiated dyspnea was associated with worse cardiac function but also with impairments in noncardiac organ function. After accounting for impairments in noncardiac organ function, dyspnea was not associated with left ventricular systolic or diastolic function but was associated with obesity, depression, pulmonary dysfunction, and extremity weakness.

**Meaning:**

The findings suggest that cardiovascular function poorly discriminates older persons with undifferentiated dyspnea from those without; therefore, dyspnea should not be assumed to represent occult heart failure in the elderly population.

## Teaser

This cross-sectional study of data from the Atherosclerosis Risk in Communities (ARIC) study quantifies the association of undifferentiated dyspnea with cardiac dysfunction in older adults.

## Introduction

Moderate to severe dyspnea is present in 17% to 25%^1,2,3^ of people older than 65 years, increasing to 30% in people 80 years and older.^3^ Self-reported dyspnea predicts incident heart failure (HF) and mortality in the general population^4^ independent of prevalent cardiopulmonary diseases and is associated with worse quality of life^5^ and greater health care use^2^ in late life. Although common, determining its cause is particularly challenging in older adults who have multiple chronic comorbidities and reduced physiological reserve in multiple organs. While dyspnea may be caused by impairments in several organ systems, it is a key symptom of HF, including HF with preserved left ventricular ejection fraction (HFpEF), the most common HF phenotype in the elderly population.^6^ Furthermore, left ventricular (LV) diastolic dysfunction, a key contributor to HFpEF, increases with age and is present in up to 50% of people older than 65 years.^7,8^ A 2010 study by Penicka et al^9^ in patients with unexplained dyspnea referred for invasive hemodynamic evaluation identified elevated LV end diastolic pressure and stiffness consistent with HFpEF in most patients. However, studies of the association of LV diastolic dysfunction with dyspnea in broader community-based samples are conflicting,^1,10,11^ and the extent to which dyspnea in late life is associated with undiagnosed HFpEF is undocumented, to our knowledge.

We hypothesized that a significant proportion of undifferentiated dyspnea in late life is associated with subclinical cardiac dysfunction, as evidenced by alterations in LV structure and impairments in LV diastolic function and systolic deformation, features commonly observed in HFpEF.^12,13,14^ We leveraged the comprehensive phenotyping of elderly participants in the Atherosclerosis Risk in Communities (ARIC) study to quantify the impairments of cardiovascular and noncardiovascular organ function that characterize undifferentiated dyspnea in older adults. Specifically, we aimed to determine the extent to which cardiovascular, pulmonary, and noncardiopulmonary organ dysfunction may distinguish participants reporting moderate or severe dyspnea from those with no or mild dyspnea.

## Methods

### Study Population

The ARIC study is an ongoing population-based cohort study that enrolled 15 792 participants from 4 communities in the United States (in North Carolina, Mississippi, Minnesota, and Maryland) from 1987 to 1989.^15^ The analysis included 5943 surviving participants (aged ≥65 years) who underwent echocardiography and dyspnea assessment at the fifth study visit (from 2011 to 2013). For analyses of dyspnea among people without a clear etiology, we excluded participants with conditions commonly associated with dyspnea, including diagnosed HF (n = 934), diagnosed chronic obstructive pulmonary disease (COPD) (n = 470), morbid obesity (body mass index [BMI; calculated as weight in kilograms divided by height in meters squared], ≥40; n = 150), and severe kidney dysfunction (estimated glomerular filtration rate [eGFR] <30 mL/min/1.73 m^2^; n = 47). After these exclusions, 4342 participants were included in our analysis. Analyses were conducted from October 2017 to June 2018.

The ARIC study was approved by institutional review boards from each site, and all participants provided written informed consent. This study followed the Strengthening the Reporting of Observational Studies in Epidemiology (STROBE) reporting guideline.

### Definition of Dyspnea

Dyspnea was assessed at visit 5 using the modified Medical Research Council scale (mMRC),^16^ graded on a scale of 0 to 4, with 0 indicating a positive answer to “Are you troubled by shortness of breath except on strenuous exertion?”; 1, a positive answer to “Are you short of breath when hurrying on the level or walking up a slight hill?”; 2, a positive answer to “Do you have to walk slower than most people on the level because of breathlessness? Do you have to stop after a mile or so (or after half an hour) on the level at your own pace?”; 3, a positive answer to “Do you have to stop for breath after walking about 100 yards (or after a few minutes) on the level?”; and 4, a positive answer to “Are you too breathless to leave the house, or breathless after undressing?” Participants were categorized as having no (mMRC score = 0), mild (mMRC score = 1), moderate (mMRC score = 2 or 3), or severe (mMRC score = 4) dyspnea.^1,3,17^

### Clinical Characteristics

Prevalence of HF in the ARIC study at visit 5 was based on physician committee–adjudicated HF hospitalizations occurring since 2005 (as previously published^18^); *International Classification of Diseases, Ninth Revision, Clinical Modification* code 428 for hospitalizations prior to 2005;^19^ or HF self-report at visits 3 through 5 or on annual follow-up telephone calls. For adjudicated hospitalizations, centrally trained and certified physicians adjudicated the HF diagnosis as definite, possible, or chronic HF.^18^ History of COPD was ascertained based on participants responding affirmatively to either of the following standardized questions: “Has a doctor ever told you that you had emphysema or chronic obstructive pulmonary disease (also called COPD)?” or “Has a doctor ever told you that you had chronic bronchitis?” Questions were administered at study visits and annual follow-up telephone calls.^20,21^

Hypertension, diabetes, and smoking status were defined as previously described.^22^ Coronary disease (ie, myocardial infarction or coronary intervention) and stroke were ascertained through ongoing ARIC study surveillance of deaths and hospitalizations and through annual telephone interviews and were centrally adjudicated as previously described.^23,24^ Edema was defined as the presence of bilateral pitting leg edema when the participant was lying flat. Body mass index was calculated from measured weight and standing height, with obesity defined as BMI of 30 or more. Participants who reported dyspnea were classified as potentially having HFpEF based on the European Society of Cardiology (ESC) guidelines^25^ if the following were present: (1) a sign of HF (ie, edema); (2) N-terminal fragment of the prohormone brain natriuretic peptide (NT-proBNP) higher than 125 pg/mL; and (3) an abnormal echocardiographic marker of structure (ie, LV hypertrophy or left atrial [LA] enlargement) or function (ie, abnormal Doppler diastolic function indices of early diastolic myocardial velocity [e′] or ratio of early mitral inflow velocity [E] to e′).

### Assessment of Cardiovascular and Noncardiovascular Function

Echocardiography in the ARIC study at visit 5 has been previously described.^26^ Briefly, all studies were acquired using uniform imaging equipment and acquisition protocol. All quantitative measures were performed in a dedicated echocardiography reading center, blinded to clinical information and in accordance with the recommendations of the American Society of Echocardiography.^27,28^ Pulmonary artery systolic pressure was estimated from Doppler echocardiography tricuspid regurgitation jet peak velocity.^26^ Arterial stiffness was assessed by carotid-femoral pulse wave velocity.^29^ Two cardiac biomarkers known to be associated with HF risk, high-sensitivity troponin T, and NT-proBNP, were assessed.^14^

Spirometry was performed at visit 5 following the American Thoracic Society quality criteria,^30^ using a dry SensorMed 827 Spirometer (Ohio Medical), connected to analysis software (OMI). Predicted reference values were derived from the Third National Health and Nutrition Examination Survey^31^ equations (Table 1). Kidney function was measured using eGFR from the Chronic Kidney Disease Epidemiology Collaboration equation.^32^ Anemia was assessed using hemoglobin levels.^33^ Lower extremity function was assessed using the Short Physical Performance Battery.^34^ Upper extremity function was assessed as the maximum handgrip isometric effort from 2 attempts using a handheld dynamometer.^35^ Depression was assessed using the Center for Epidemiologic Studies Depression 11-item questionnaire.^36^

**Table 1.** Cardiac and Noncardiac Metrics and Associated Definitions for Dysfunction Used in Analysis

Function Metric	Metric	Abnormal Measure	
		Men	Women
Cardiovascular function			
LV structure	LV mass index, g/m^2^	>96.1	>83
LV systolic function^a^	Ejection fraction, %	<60	<59
	Longitudinal strain, %	<−16	<−16
	Circumferential strain, %	<−23	<−23
LV diastolic function^b^	Left atrial diameter, cm	>4.0	>3.7
	Left atrial volume index, mL/m^2^	>31	>30
	Lateral TDI e′, cm/s	<5.4	<5.1
	Septal TDI e′, cm/s	<4.6	<4.5
	Lateral E/e′ ratio	>11.5	>13.3
	Septal E/e′ ratio	>13.3	>15.1
RV function	RV fractional area change; tricuspid annular peak systolic myocardial velocity, cm/s	Dysfunction not considered	Dysfunction not considered
Systemic arterial function	Mean arterial pressure, mm Hg; pulse pressure, mm Hg; carotid-femoral pulse wave velocity, cm/s	Dysfunction not considered	Dysfunction not considered
Pulmonary function	Restrictive ventilatory pattern, % predicted FVC	<80	<80
	Obstructive ventilatory pattern, FEV_1_/FVC ratio, %^31^	<70	<70
Pulmonary hypertension	Estimated PASP, mm Hg^c^	>32	>32
Renal function	Estimated glomerular filtration rate, mL/min.1.73 m^2^	<60	<60
Hematologic function	Hemoglobin level, g/dL^33^	<13	<12
Lower extremity function	Lower extremity Short Physical Performance Battery score (range, 0-12)^34^	≤6	≤6
Upper extremity function^35^	BMI-based handgrip strength, kg	BMI ≤24.0; handgrip strength ≤29	BMI ≤23.0; handgrip strength ≤17
		BMI 24.1-26.0; handgrip strength ≤30	BMI 23.1-26.0; handgrip strength ≤17.3
		BMI 26.1-28.0; handgrip strength ≤30	BMI 26.1-29.0; handgrip strength ≤18
		BMI >28; handgrip strength ≤32	BMI >29.1; handgrip strength ≤21
Depression score	Center for Epidemiologic Studies Depression Scale score (range, 0-22)^36^	≥9	≥9
Adiposity^37^	BMI	≥30	≥30

Abbreviations: BMI, body mass index (calculated as weight in kilograms divided by height in meters squared); E, early mitral inflow velocity; e′, early diastolic myocardial velocity; FEV_1_, forced expiratory volume in the first second of expiration; FVC, forced vital capacity; LV, left ventricle; PASP, pulmonary artery systolic pressure; RV, right ventricle; TDI, tissue Doppler velocity.

SI conversion: To convert hemoglobin to g/L, multiply by 10.

^a^ Participants with test results meeting the cutoff value for any 1 metric were considered to have LV systolic dysfunction.

^b^ Participants with test results meeting the cutoff value for any 2 metrics were considered to have LV diastolic dysfunction.

^c^ Assuming a right atrial pressure of 5 mm Hg.

Specific metrics of organ function, cutoff values for abnormal metrics, and definitions for abnormal metrics are provided in Table 1. The ARIC study–based sex-specific reference limits were used to define abnormal for echocardiographic measures.^8,14^ Cardiac dysfunctions were (1) LV hypertrophy, defined as elevated LV mass index; (2) systolic dysfunction, defined as an abnormally low LV ejection fraction, longitudinal strain, or circumferential strain; (3) diastolic dysfunction, defined as abnormal values of at least 2 of 3 diastolic metrics (ie, LA diameter or LA volume index, lateral or septal e′, and lateral or septal E/e′ ratio); and (4) pulmonary hypertension (pHTN), defined as an abnormally high tricuspid regurgitation velocity. Restrictive ventilatory pattern was defined if forced vital capacity (FVC) was less than 80% of the predicted value, and obstructive ventilatory pattern was defined as the ratio of forced expiratory volume in the first second of expiration (FEV_1_) to FVC less than 70% of the predicted value.^31^ Renal dysfunction, anemia, extremity weakness, depression, and obesity were defined using clinically accepted cutoff values, as previously used in the ARIC study analyses.^32,33,34,35,36,37^

### Statistical Analysis

Clinical characteristics and quantitative measures of organ function were initially compared among participants with no dyspnea, mild dyspnea, or moderate to severe dyspnea to assess trends across dyspnea severity using multivariable linear, logistic, or quantile regression (for nonnormally distributed continuous variables) adjusted for age, sex, and race/ethnicity. We then dichotomized participants based on no to mild dyspnea (mMRC score <2) or moderate to severe dyspnea (mMRC score ≥2), given the established prognostic value of these thresholds,^1,3,17^ and assessed their associations with each metric of organ system dysfunction. Cardiovascular dysfunction was defined as abnormal LV structure (hypertrophy), systolic dysfunction, and diastolic dysfunction. Pulmonary dysfunction was defined as obstructive ventilatory pattern or restrictive ventilatory pattern. Associations with pHTN were also assessed. Noncardiopulmonary organ dysfunction was defined as kidney dysfunction, anemia, lower extremity weakness, upper extremity weakness, depression symptoms, or obesity. Specific metrics of organ function and definitions of abnormal values used in the ARIC study are described in Table 1. The association of each dysfunction with moderate to severe dyspnea was assessed using multivariable logistic regression analysis adjusted for age, sex, and race/ethnicity. All metrics that were significantly associated with dyspnea in these models were included together in a final multivariable logistic regression model with age, sex, and race/ethnicity. Potential confounders (ie, study center, household income,^38^ and physical activity^39^) were also included in a sensitivity analysis.

We then calculated the population-attributable risk (PAR) for moderate to severe dyspnea associated with each dysfunction metric. We used the adjustment for relative risks (RRs) to account for possible confounding using the following formula: PAR = pd*_i_* × [(RR*_i_* − 1)/RR*_i_*], in which pd*_i_* indicates the proportion of total cases in the population in the *i*th exposure category and RR*_i_* is the adjusted RR for the *i*th exposure category. The PAR percentage associated with each dysfunction metric was estimated in the overall population using the odds ratio (OR) estimates derived from the multivariable model adjusted for age, sex, race/ethnicity, and all selected dysfunction metrics. To examine the association of inclusion bias with potentially nonrandom visit 5 nonattendance, we applied inverse–probability-of-attrition weighting^40^ (eAppendix in the Supplement).

We calculated the ORs with 95% CIs to determine the magnitude of the associations. Missing data represented less than 5% for all variables, except for percentage of predicted FVC (871 [20.1%]) and FEV_1_ to FVC ratio (900 [20.7%]), which were missing owing to unacceptable quality. No imputation was performed. A 2-sided *P* value less than .05 was considered statistically significant for all analyses. Statistical analysis was performed using Stata statistical software version 14.2 (StataCorp).

## Results

### Dyspnea and Cardiovascular Function

Among the 5943 participants in the ARIC study population at the fifth visit (mean [SD] age, 76.0 [5.1] years; 3439 [57.9%] women), 2159 (36.3%) reported dyspnea and 1288 participants (21.7%) reported moderate to severe dyspnea (eFigure 1 in the Supplement). Among the 4342 ARIC study participants included in this analysis (mean [SD] age, 75.9 [5.0] years; 2533 [58.3%] women) who were free of conditions clinically associated with dyspnea (ie, HF, COPD, BMI ≥40, or eGFR <30 mL/min/1.73 m^2^), 1173 (27.0%) reported dyspnea and 574 (13.2%) reported dyspnea that was moderate to severe (eFigure 1 in the Supplement). Moderate to severe dyspnea was present in 574 participants (13.2%) and was associated with LV hypertrophy (OR, 1.53; 95% CI, 1.25-1.87; *P* < .001) and LV diastolic (OR, 1.46; 95% CI, 1.20-1.78; *P* < .001) and systolic (OR, 1.28; 95% CI, 1.05-1.56; *P* = .02) dysfunction. Moderate to severe dyspnea was also associated with obstructive (OR, 1.59; 95% CI, 1.28-1.99; *P* < .001) and restrictive (OR, 2.56; 95% CI, 1.99-3.27; *P* < .001) findings on spirometry, renal impairment (OR, 1.32; 95% CI, 1.08-1.61; *P* = .01), anemia (OR, 1.72; 95% CI, 1.39-2.12; *P* < .001), lower (OR, 2.77; 95% CI, 2.18-3.51; *P* < .001) and upper (OR, 1.82; 95% CI, 1.49-2.23; *P* < .001) extremity weakness, depression (OR, 3.01; 95% CI, 2.24-4.25; *P* < .001), and obesity (OR, 2.35; 95% CI, 1.95-2.83; *P* < .001). After accounting for these, moderate to severe dyspnea was associated with LV hypertrophy (OR, 1.30; 95% CI, 1.01-1.67; *P* = .04) and was not associated with systolic or diastolic function. Older age, female sex, and black race were associated with increased dyspnea severity (Table 2). After adjusting for these, dyspnea was also associated with several cardiovascular comorbidities, higher prevalence of bilateral lower extremity edema, and higher concentrations of NT-proBNP and high-sensitivity troponin T (Table 2).

**Table 2.** Clinical Characteristics, and Metrics of Cardiovascular and Noncardiovascular Function by Severity of Reported Dyspnea

Clinical Characteristic	Dyspnea, Mean (SD)			*P* Value for Trend^a^
	None	Mild	Moderate to Severe	
Total, No. (%)	3169 (73.0)	599 (13.8)	574 (13.2)	NA
Demographic characteristics				
Age, y	75.4 (4.9)	76.4 (5.0)	77.4 (5.3)	<.001
Female sex, No. (%)	1764 (55.7)	397 (66.3)	372 (64.8)	<.001
Black race, No. (%)	563 (17.8)	116 (19.4)	169 (29.4)	<.001
Cardiovascular disease and risk factors, No. (%)				
Hypertension	2459 (77.6)	517 (86.3)	510 (88.9)	<.001
Diabetes	957 (30.2)	224 (37.4)	253 (44.1)	<.001
Current smoker	149 (4.7)	35 (5.9)	38 (6.7)	.003
Former smoker	1473 (49.7)	281 (50.3)	261 (49.9)	.12
Coronary heart disease	276 (8.9)	58 (9.9)	67 (11.9)	.002
Stroke	59 (1.9)	19 (3.2)	26 (4.5)	<.001
Markers of heart failure, No. (%)				
Lower extremity edema	345 (11.1)	96 (16.2)	122 (21.7)	<.001
Diuretic use	87 (2.8)	38 (6.4)	45 (7.9)	<.001
NT-proBNP, median (IQR), pg/mL	108 (58-208)	136 (71-256)	160 (81-318)	<.001
High-sensitivity to troponin T, median (IQR), ng/L	10.0 (7.0-14.0)	10.0 (7.0-15.0)	12.0 (9.0-18.0)	<.001
LV structure				
Mass index, g/m^2^	77 (18)	79 (18)	80 (20)	<.001
End-diastolic volume index, mL/m^2^	43.9 (10.2)	42.1 (9.3)	42.4 (9.3)	.06
Mean wall thickness, cm	0.97 (0.13)	0.99 (0.13)	1.01 (0.14)	<.001
Relative wall thickness, cm	0.42 (0.07)	0.43 (0.08)	0.44 (0.09)	<.001
LV systolic function, %				
Ejection fraction	65.8 (5.8)	65.8 (6.4)	65.6 (6.4)	.26
Longitudinal strain	−18.2 (2.4)	−18.0 (2.5)	−17.8 (2.6)	<.001
Circumferential strain	−28.0 (3.7)	−27.9 (4.1)	−27.5 (3.6)	.002
LV diastolic function				
LA diameter, cm	3.49 (0.49)	3.52 (0.45)	3.56 (0.54)	<.001
LA volume index, mL/m^2^	25.1 (8.3)	26.2 (7.8)	26.4 (8.8)	.001
Lateral e′, cm/s	7.2 (2.0)	6.9 (1.8)	6.8 (2.1)	.05
Septal e′, cm/s	5.8 (1.5)	5.5 (1.3)	5.5 (1.6)	.001
E/e′ lateral	9.8 (3.6)	10.3 (3.5)	10.6 (4.0)	<.001
E/e′ septal	11.8 (3.9)	12.6 (3.8)	13.0 (4.7)	<.001
Right ventricle function and pulmonary hemodynamics				
Fractional area change	0.52 (0.08)	0.53 (0.08)	0.52 (0.08)	.97
Tricuspid annular peak systolic myocardial velocity, cm/s	11.9 (2.8)	11.7 (3.0)	11.7 (3.0)	.52
Estimated pulmonary artery systolic pressure, mm Hg	27 (5)	29 (7)	29 (6)	<.001
Systemic arterial, mean (SD)				
Systolic pressure, mm Hg	130 (17)	131 (19)	131 (18)	.96
Diastolic pressure, mm Hg	67 (10)	67 (11)	66 (11)	.75
Pulse pressure, mm Hg	63 (14)	64 (15)	65 (14)	.76
Carotid-femoral pulse wave velocity, cm/s	1154 (337)	1170 (293)	1213 (348)	.37
Pulmonary				
% Of predicted FEV_1_	98.3 (18.5)	91.9 (19.1)	89.4 (22.4)	<.001
% Of predicted FVC	99 (19)	94 (18)	91 (22)	<.001
FEV_1_/FVC ratio (%)	73.2 (7.4)	72.9 (8.4)	71.3 (10.0)	<.001
Estimated glomerular filtration rate, mL/min/1.73 m^2^	71.9 (15)	69.8 (16)	68.5 (17)	<.001
Hemoglobin, g/dL	13.5 (1.3)	13.3 (1.4)	12.9 (1.5)	<.001
Physical function)				
Short Physical Performance Battery score	9.9 (2.1)	9.4 (2.2)	8.1 (2.8)	<.001
Grip strength, kg	30.2 (10.5)	27.7 (9.7)	26.9 (9.4)	<.001
Center for Epidemiologic Studies Depression scale score	2.4 (2.5)	3.5 (2.9)	4.1 (3.3)	<.001
Body mass index^b^	27.3 (4.3)	29.2 (4.5)	29.6 (4.9)	<.001

Abbreviations: E, early mitral inflow velocity; e′, early diastolic myocardial velocity; FEV_1_, forced expiratory volume in 1 second; FVC, forced vital capacity; IQR, interquartile range; LA, left atrial; LV, left ventricle; NA, not applicable; NT-proBNP, N-terminal fragment of the prohormone brain natriuretic peptide.

SI conversion factor: To convert hemoglobin to grams per liter, multiply by 10.

^a^ Adjusted *P* values for age, sex, and race/ethnicity to all variables, except for age, female sex, and black race. Participants did not have heart failure, chronic obstructive pulmonary disease, body mass index ≥40, or estimated glomerular filtration rate <30mL/min/1.73 m^2^.

^b^ Calculated as weight in kilograms divided by height in meters squared.

Greater dyspnea severity was associated with higher LV mass index and greater wall thickness but was not associated with LV cavity size (Table 2). Although no significant difference was noted in LV ejection fraction, longitudinal and circumferential strains decreased across dyspnea categories. Echocardiographic markers of LV filling pressure (ie, E/e′ or LA size), LV relaxation velocities (ie, e′), and pulmonary artery systolic pressure worsened across dyspnea categories, although no significant differences were noted in right ventricular function. Systemic arterial measures were not associated with dyspnea. Among participants with undifferentiated moderate to severe dyspnea, 58 (10.1%) met all ESC criteria for potential HFpEF using ARIC study cutoff points, and 76 (13.2%) met the criteria for potential HFpEF when ESC guideline cutoff points for echocardiographic measures were substituted for ARIC study–based cutoff points.

### Dyspnea and Noncardiovascular Organ Function

**Figure 1.**
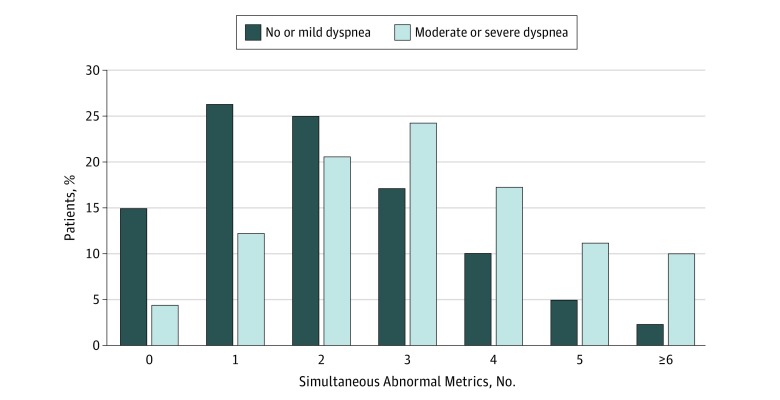
Number of Abnormal Metrics of Cardiac and Noncardiac Organ Function Among Participants With Moderate to Severe vs No or Mild Dyspnea After adjusting for age, sex, and race/ethnicity, participants with moderate to severe dyspnea had significantly more simultaneous dysfunctions (median [interquartile range], 3 [2-4]) than those with no or mild dyspnea (median [interquartile range], 2 [1-3]) (*P* < .001).

Spirometric measures worsened across dyspnea severity categories (Table 2), as did measures of noncardiopulmonary organ function, including eGFR, hemoglobin, upper and lower extremity physical function, depressive symptoms, and BMI (Table 2). Participants with moderate to severe dyspnea also demonstrated a greater number of abnormal organ function metrics compared with those with no to mild dyspnea (Figure 1).

**Figure 2.**
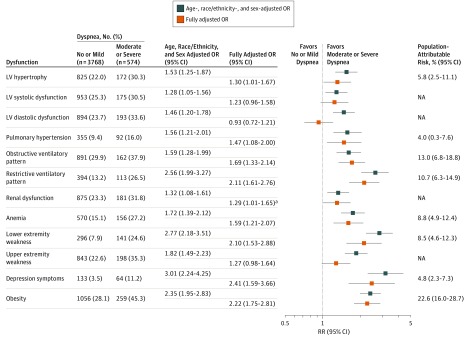
Adjusted Associations of Cardiovascular and Noncardiovascular Organ Dysfunction With Undifferentiated Dyspnea in the Elderly Population The fully adjusted model was adjusted for age, sex, race/ethnicity, and all listed metrics of organ dysfunction. LV indicates left ventricle; NA, not applicable; OR, odds ratio; RR, relative risk. ^a^*P* = .05.

In models adjusted for participant demographic characteristics, all cardiovascular and noncardiovascular dysfunction metrics were associated with higher odds of having moderate to severe dyspnea (Figure 2). When adjusting for all metrics simultaneously, obesity (OR, 2.22; 95% CI, 1.75-2.81; *P* < .001), restrictive spirometric pattern (OR, 2.11; 95% CI, 1.61-2.76; *P* < .001), lower extremity weakness (OR, 2.10; 95% CI, 1.53-2.88; *P* < .001), and depression (OR, 2.41; 95% CI, 1.59-3.66; *P* < .001) were associated with moderate to severe dyspnea. Pulmonary hypertension (OR, 1.47; 95% CI, 1.08-2.00; *P* = .02), obstructive ventilatory pattern (OR, 1.69; 95% CI, 1.33-2.14; *P* < .001), and anemia (OR, 1.59; 95% CI, 1.21-2.07; *P* = .001) were also associated with moderate to severe dyspnea (Figure 2). Aside from an association with LV hypertrophy (OR, 1.30; 95% CI, 1.01-1.67; *P* = .04), LV systolic (OR, 1.23; 95% CI, 0.96-1.58; *P* = .10) and diastolic (OR, 0.93; 95% CI, 0.72-1.21; *P* = .59) dysfunction were not independently associated with moderate to severe dyspnea. These findings were unchanged when cardiac metrics (ie, LV hypertrophy, systolic dysfunction, and diastolic dysfunction) were introduced separately into multivariable models to account for possible collinearity (Table 3). Similar findings were also noted in sensitivity analyses excluding lower extremity dysfunction and depression, in analyses comparing no dyspnea with moderate to severe dyspnea (eFigure 2 and eFigure 3 in the Supplement), and in analyses using inverse–probability-of-attrition weighting to account for potential attendance bias (eTables 1-4 in the Supplement). To assess the potential association of obesity and restrictive ventilatory pattern in the results, additional sensitivity analyses of the fully adjusted model were performed, excluding either restrictive ventilatory pattern or obesity while keeping the other in the model. These analyses demonstrated results consistent with the primary analysis (Table 3). Similar findings were observed in analyses additionally adjusted for field center, household income, and physical activity (eTable 5 and eTable 6 in the Supplement). Obesity accounted for the highest PAR of moderate to severe dyspnea (22.6%), while the PAR associated with LV hypertrophy was 5.8% (Figure 2).

**Table 3.** Sensitivity Analysis of Association of Moderate to Severe Dyspnea With Cardiovascular and Noncardiovascular Dysfunctions Accounting for Potential Reverse Causality or Colinearity

Dysfunction	Odds Ratio (95% CI)					
	Sensitivity Analysis 1^a^			Sensitivity Analysis 2^b^		
	Model 1	Model 2	Model 3	Model 4	Model 5	Model 6
LV hypertrophy	1.33 (1.04-1.70)	1.35 (1.06-1.73)	1.38 (1.08-1.77)	1.31 (1.03-1.68)	NA	NA
LV systolic dysfunction	1.25 (0.98-1.60)	1.24 (0.97-1.59)	1.22 (0.95-1.56)	NA	1.28 (1.00-1.63)	NA
LV diastolic dysfunction	0.97 (0.76-1.25)	0.99 (0.77-1.27)	0.97 (0.75-1.26)	NA	NA	1.02 (0.79-1.31)
Pulmonary hypertension	1.46 (1.08-1.98)	1.56 (1.15-2.11)	1.47 (1.08-1.99)	1.44 (1.06-1.96)	1.47 (1.08-2.01)	1.44 (1.06-1.97)
Obstructive pattern	1.71 (1.35-2.15)	1.71 (1.35-2.17)	1.53 (1.21-1.94)	1.69 (1.33-2.15)	1.70 (1.34-2.16)	1.71 (1.34-2.17)
Restrictive pattern	2.14 (1.64-2.80)	NA	2.30 (1.76-3.01)	2.13 (1.62-2.80)	2.15 (1.64-2.81)	2.18 (1.66-2.85)
Renal dysfunction	1.27 (0.99-1.61)	1.30 (1.02-1.66)	1.35 (1.05-1.72)	1.29 (1.01-1.66)	1.28 (1.00-1.64)	1.29 (1.01-1.65)
Anemia	1.67 (1.28-2.17)	1.61 (1.24-2.09)	1.51 (1.16-1.97)	1.57 (1.20-2.05)	1.62 (1.24-2.11)	1.60 (1.23-2.10)
Lower extremity weakness	NA	2.16 (1.59-2.95)	2.15 (1.57-2.94)	2.10 (1.53-2.88)	2.12 (1.55-2.90)	2.13 (1.55-2.92)
Upper extremity weakness	NA	1.32 (1.03-1.70)	1.44 (1.12-1.86)	1.27 (0.98-1.64)	1.27 (0.98-1.64)	1.27 (0.99-1.64)
Depression symptoms	NA	2.30 (1.52-3.46)	2.49 (1.64-3.76)	2.41 (1.59-3.66)	2.48 (1.64-3.75)	2.50 (1.65-3.78)
Obesity	2.35 (1.87-2.95)	2.27 (1.80-2.86)	NA	2.21 (1.74-2.79)	2.28 (1.80-2.88)	2.27 (1.80-2.87)

Abbreviations: LV, left ventricle; NA, not applicable.

^a^ Sensitivity analysis 1 does not adjust for assigned variables, given the possibility of reverse causation. Model 1 adjusts for extremity weakness or depression; model 2, restrictive ventilatory pattern; and model 3, obesity.

^b^ Sensitivity analysis 2 adjusts for each cardiovascular metric individually to account for the possible impact of collinearity of cardiovascular measures. Model 4 adjusts for LV hypertrophy; model 5, LV systolic function; and model 6, LV diastolic function.

## Discussion

In this large, biracial, community-based cohort, the prevalence of undifferentiated moderate to severe dyspnea was 13.2% among older adults without diagnosed HF, COPD, BMI of 40 or higher, or eGFR less than 30 mL/min/1.73 m^2^. Greater dyspnea severity was associated with lower extremity edema, higher LV mass index, worse LV systolic and diastolic function, and higher concentrations of biomarkers of myocardial stress and injury—all findings suggestive of possible occult HFpEF. However, greater dyspnea was also associated with pulmonary and other noncardiovascular organ dysfunction, and after accounting for these, cardiovascular metrics poorly discriminated participants with moderate to severe dyspnea from those without dyspnea. In contrast, obesity, restrictive ventilatory pattern, pHTN, lower extremity weakness, and depressive symptoms did discriminate participants with moderate or severe dyspnea, with the PAR associated with obesity alone of 23%. These findings support the multifactorial nature of dyspnea in the elderly population and highlight that undifferentiated dyspnea in the elderly population should not be assumed to be caused by occult HF.

The prevalence of dyspnea, which is known to increase with age,^1,3^ in our study was comparable with other cohorts of similar age.^1,2^ Consistent with prior studies, moderate to severe dyspnea was associated with cardiovascular comorbidities, including hypertension, diabetes, and obesity, and with several cardiovascular and noncardiovascular physiological impairments. Undifferentiated dyspnea was associated with greater LV mass and worse LV diastolic and systolic function. The association of moderate to severe dyspnea with LV hypertrophy is consistent with the known association of LV hypertrophy with lower functional capacity^41,42^ but contrasts with findings from the Multi-ethnic Study of Atherosclerosis,^17^ although that was a younger cohort (mean [SD] age, 62 [10] years) with an appreciably lower hypertension prevalence (42%). However, while this association persisted in our study in fully adjusted models, the OR for LV hypertrophy was lower.

Data on the association of dyspnea with diastolic dysfunction have been conflicting, with smaller studies (<300 elderly participants) not observing a significant association.^10,11^ However, a larger 2016 study by Miner et al^1^ analyzing 4413 participants 65 years and older in the Cardiovascular Health Study found that both LV diastolic (the ratio of peak velocity blood flow from gravity in early diastole to peak velocity flow in late diastole caused by atrial contraction) and systolic (LVEF <54%) dysfunction were associated with self-reported dyspnea.^1^ Our study supported these findings with the association of dyspnea with more sensitive markers of diastolic (ie, tissue Doppler imaging e′, E/e′ ratio, and LA size) and systolic function (longitudinal and circumferential strain). However, these LV functional measures did not discriminate participants with dyspnea from those without dyspnea after accounting for noncardiovascular organ function in our analysis. Pulmonary hypertension, a potential manifestation of elevated LV filling pressure, did demonstrate a modest independent association with moderate to severe dyspnea. However, the causes underlying pHTN in this context are likely multifactorial, including pulmonary disease,^43^ obesity,^44^ and intrinsic age-related alterations in the pulmonary vasculature.^45^

The most notable result of our analysis, which was contrary to our a priori hypothesis, was that cardiovascular measures had only a modest association with dyspnea when accounting for impairments in noncardiovascular organ systems. It is likely that moderate to severe dyspnea represent the transition to stage C HF in a subset of participants, and approximately 10% met ESC criteria for HFpEF. However, our findings highlight that many factors contribute to dyspnea in elderly people, with only a modest independent association with cardiovascular function. Concordant with this complexity, several studies have posited that dyspnea in the elderly population should be interpreted as a broader clinical syndrome, representing an overlap of several different chronic diseases.^1,2,3^

Dyspnea has consistently been associated with spirometric abnormalities.^1,3,17,46^ Notably, compared with an obstructive pattern, we observed a higher odds ratio associated with a restrictive pattern. While low or decreasing FVC may represent pulmonary congestion, this association persisted after accounting for LV function and filling pressure, making occult HF unlikely. Potential mechanisms in this cohort of elderly people include obesity, sarcopenia, and kyphoscoliosis.^47^ Obesity demonstrated a significant association with moderate to severe dyspnea. Obesity may potentiate impairments in cardiovascular,^37,48^ pulmonary, and skeletal muscle function.^49^ However, in our study and others,^1,2,46^ obesity was associated with dyspnea independent of these, suggesting a primary association of obesity itself with dyspnea, possibly owing to a heightened perception of breathlessness.^50^ The independent associations of lower extremity weakness,^51,52^ anemia, and depression with dyspnea further highlight the range of pathophysiological and psychophysiological mechanisms contributing to dyspnea in elderly people.

### Limitations

Several limitations should be noted. The cross-sectional design precludes causal determination. Reverse causation is possible for the association of dyspnea with some exposure variables, particularly extremity muscle function, depression, and obesity. The impairments considered in this analysis interacted with and may have potentiated each other in complex ways,^53^ such that modeling each as independently associated with dyspnea may be an oversimplification. However, the modest magnitude of associations of cardiac measures with dyspnea in multivariate analysis supports their weak associations compared with other factors and persisted in multiple sensitivity analyses. Only a subset of ARIC study participants alive at the time of the fifth visit chose to attend, potentially introducing bias. However, sensitivity analysis with inverse–probability-of-attrition weighting showed similar results as the primary analysis. Although resting echocardiography is the most commonly used test to assess for cardiac dysfunction in dyspnea or suspected HFpEF, cardiopulmonary exercise testing with invasive hemodynamic monitoring may be required to detect cardiac dysfunction in some patients.^54^ We only designated potential HFpEF based on ESC criteria, as HFpEF is a clinical diagnosis best confirmed by physician evaluation. A limitation of dichotomizing exposure variables is that the severity of dysfunction for different systems may differ among systems. For example, the magnitude of pHTN at the cutoff used in this analysis may differ from the magnitude of systolic dysfunction captured by the cutoff values used for those measures. While commonly used in epidemiological studies, self-report of COPD may underestimate airflow limitation compared with a direct measure but demonstrates high specificity for detecting COPD and identifying individuals with more severe disease.^55^ Additionally, we excluded 150 participants with BMI of 40 or greater, given the potential primary contribution of morbid obesity to dyspnea, but HFpEF may also occur in this context.

## Conclusions

In this large, biracial, community-based cohort of elderly people, the prevalence of undifferentiated dyspnea was 27% among persons who had not been diagnosed with HF or with COPD, morbid obesity, or severe kidney disease and was moderate to severe in 13% of the cohort. Impairments of cardiac structure and function were more frequent among those with undifferentiated dyspnea, as were pulmonary and noncardiovascular organ dysfunction. When considered in the context of other organ systems potentially contributing to dyspnea, measures of cardiovascular function poorly discriminated elderly people with dyspnea from those without dyspnea. These findings highlight that dyspnea in the elderly population is multifactorial and likely represents a broader clinical syndrome associated with overlap of several different chronic diseases, including particularly prominent contributions from obesity, restrictive ventilatory pattern, extremity weakness, and depression. Therefore, moderate to severe dyspnea in the elderly population should not be assumed to be associated with HFpEF without further diagnostic investigations.

## Notes

Supplement. **eFigure 1.** Prevalence of Dyspnea Among Atherosclerosis Risk in Communities (ARIC) Study Participants at the Fifth Study Visit **eFigure 2.** Histogram of the Number of Abnormal Metrics of Cardiac and Noncardiac Organ Function Among Participants With No Dyspnea vs Moderate to Severe Dyspnea **eFigure 3.** Adjusted Associations of Cardiovascular and Noncardiovascular Organ Dysfunction With Moderate to Severe Compared With No Dyspnea **eAppendix.** Methods for Inverse–Probability-of-Attrition Weighting Analysis **eTable 1.** Clinical Characteristics by Dyspnea Severity Using Inverse–Probability-of-Attrition Weighting **eTable 2.** Measures of Cardiovascular Function by Dyspnea Severity Using Inverse–Probability-of-Attrition Weighting **eTable 3.** Measures of Noncardiovascular Function by Dyspnea Severity Using Inverse–Probability-of-Attrition Weighting **eTable 4.** Prevalence of Cardiovascular and Noncardiovascular Dysfunction by Dyspnea Severity Using Inverse–Probability-of-Attrition Weighting **eTable 5.** Clinical Characteristics and Metrics of Cardiovascular and Noncardiovascular Function by Severity of Reported Dyspnea, Adjusted for Age, Sex, Race, Household Income, Study Center, and Leisure-Time Physical Activity **eTable 6.** Associations of Cardiovascular and Noncardiovascular Organ Dysfunctions With Moderate to Severe Compared With No to Mild Dyspnea Adjusted for Socioeconomic Characteristics
