# Supplementary material for: Association of Undifferentiated Dyspnea in Late Life With Cardiovascular and Noncardiovascular Dysfunction: A Cross-sectional Analysis From the ARIC Study
Source: JAMA Netw Open. 2019 Jun 14;2(6):e195321. doi: 10.1001/jamanetworkopen.2019.5321 (PMC6575149; doi:10.1001/jamanetworkopen.2019.5321)
Supplement: Supplement. — eFigure 1. Prevalence of Dyspnea Among Atherosclerosis Risk in Communities (ARIC) Study Participants at the Fifth Study Visit eFigure 2. Histogram of the Number of Abnormal Metrics of Cardiac and Noncardiac Organ Function Among Participants With No Dyspnea vs Moderate to Severe Dyspnea eFigure 3. Adjusted Associations of Cardiovascular and Noncardiovascular Organ Dysfunction With Moderate to Severe Compared With No Dyspnea eAppendix. Methods for Inverse–Probability-of-Attrition Weighting Analysis eTable 1. Clinical Characteristics by Dyspnea Severity Using Inverse–Probability-of-Attrition Weighting eTable 2. Measures of Cardiovascular Function by Dyspnea Severity Using Inverse–Probability-of-Attrition Weighting eTable 3. Measures of Noncardiovascular Function by Dyspnea Severity Using Inverse–Probability-of-Attrition Weighting eTable 4. Prevalence of Cardiovascular and Noncardiovascular Dysfunction by Dyspnea Severity Using Inverse–Probability-of-Attrition Weighting eTable 5. Clinical Characteristics and Metrics of Cardiovascular and Noncardiovascular Function by Severity of Reported Dyspnea, Adjusted for Age, Sex, Race, Household Income, Study Center, and Leisure-Time Physical Activity eTable 6. Associations of Cardiovascular and Noncardiovascular Organ Dysfunctions With Moderate to Severe Compared With No to Mild Dyspnea Adjusted for Socioeconomic Characteristics [file jamanetwopen-2-e195321-s001.pdf]

Supplementary Online Content

Ramalho SHR, Santos M, Claggett B, et al. Association of undifferentiated dyspnea in late life with cardiovascular and noncardiovascular dysfunction: a cross-sectional analysis from the ARIC study. *JAMA Netw Open*. 2019;2(6):e195321.
doi:10.1001/jamanetworkopen.2019.5321

eFigure 1. Prevalence of Dyspnea Among Atherosclerosis Risk in Communities (ARIC) Study Participants at the Fifth Study Visit
eFigure 2. Histogram of the Number of Abnormal Metrics of Cardiac and Noncardiac Organ Function Among Participants With No Dyspnea vs Moderate to Severe Dyspnea
eFigure 3. Adjusted Associations of Cardiovascular and Noncardiovascular Organ Dysfunction With Moderate to Severe Compared With No Dyspnea
eAppendix. Methods for Inverse–Probability-of-Attrition Weighting Analysis
eTable 1. Clinical Characteristics by Dyspnea Severity Using Inverse–Probability-of-Attrition Weighting
eTable 2. Measures of Cardiovascular Function by Dyspnea Severity Using Inverse–Probability-of-Attrition Weighting
eTable 3. Measures of Noncardiovascular Function by Dyspnea Severity Using Inverse–Probability-of-Attrition Weighting
eTable 4. Prevalence of Cardiovascular and Noncardiovascular Dysfunction by Dyspnea Severity Using Inverse–Probability-of-Attrition Weighting
eTable 5. Clinical Characteristics and Metrics of Cardiovascular and Noncardiovascular Function by Severity of Reported Dyspnea Adjusted for Age, Sex, Race, Household Income, Study Center, and Leisure-Time Physical Activity
eTable 6. Associations of Cardiovascular and Noncardiovascular Organ Dysfunctions With Moderate to Severe Compared With No to Mild Dyspnea Adjusted for Socioeconomic Characteristics

This supplementary material has been provided by the authors to give readers additional information about their work.

© 2019 Ramalho SHR et al. *JAMA Network Open.*

eFigure 1. Prevalence of Dyspnea Among Atherosclerosis Risk in Communities (ARIC) Study Participants at the Fifth Study Visit

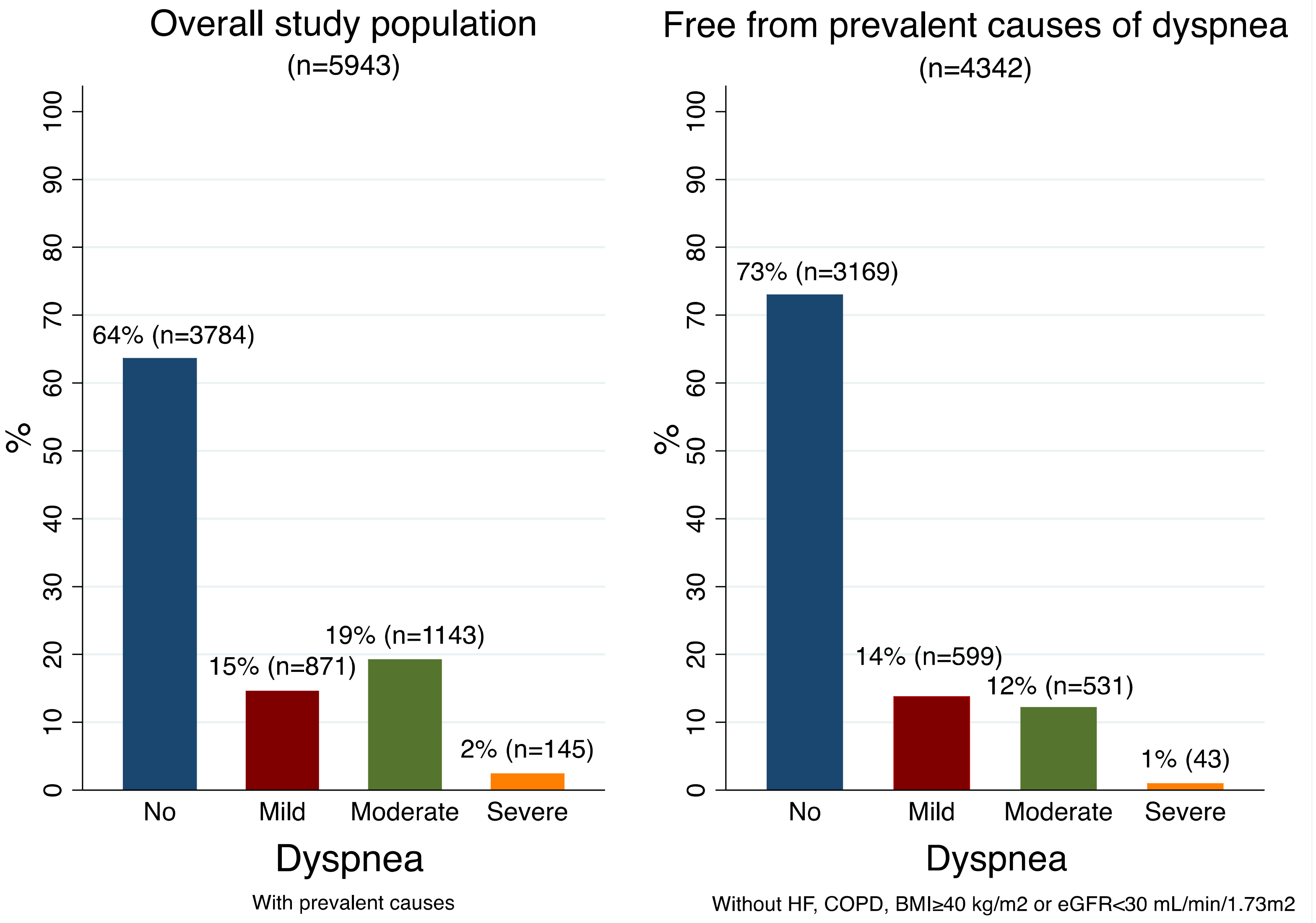

Legend: HF: heart failure; COPD: chronic pulmonary obstructive disease; BMI: body mass index; eGFR: estimated glomerular filtration rate. No dyspnea: mMRC=0, Mild: mMRC=1; Moderate: mMRC=2 and 3; Severe: mMRC=4

© 2019 Ramalho SHR et al. *JAMA Network Open.*

eFigure 2. Histogram of the Number Of Abnormal Metrics of Cardiac And Noncardiac Organ Function Among Participants With No Dyspnea Versus Moderate to Severe Dyspnea

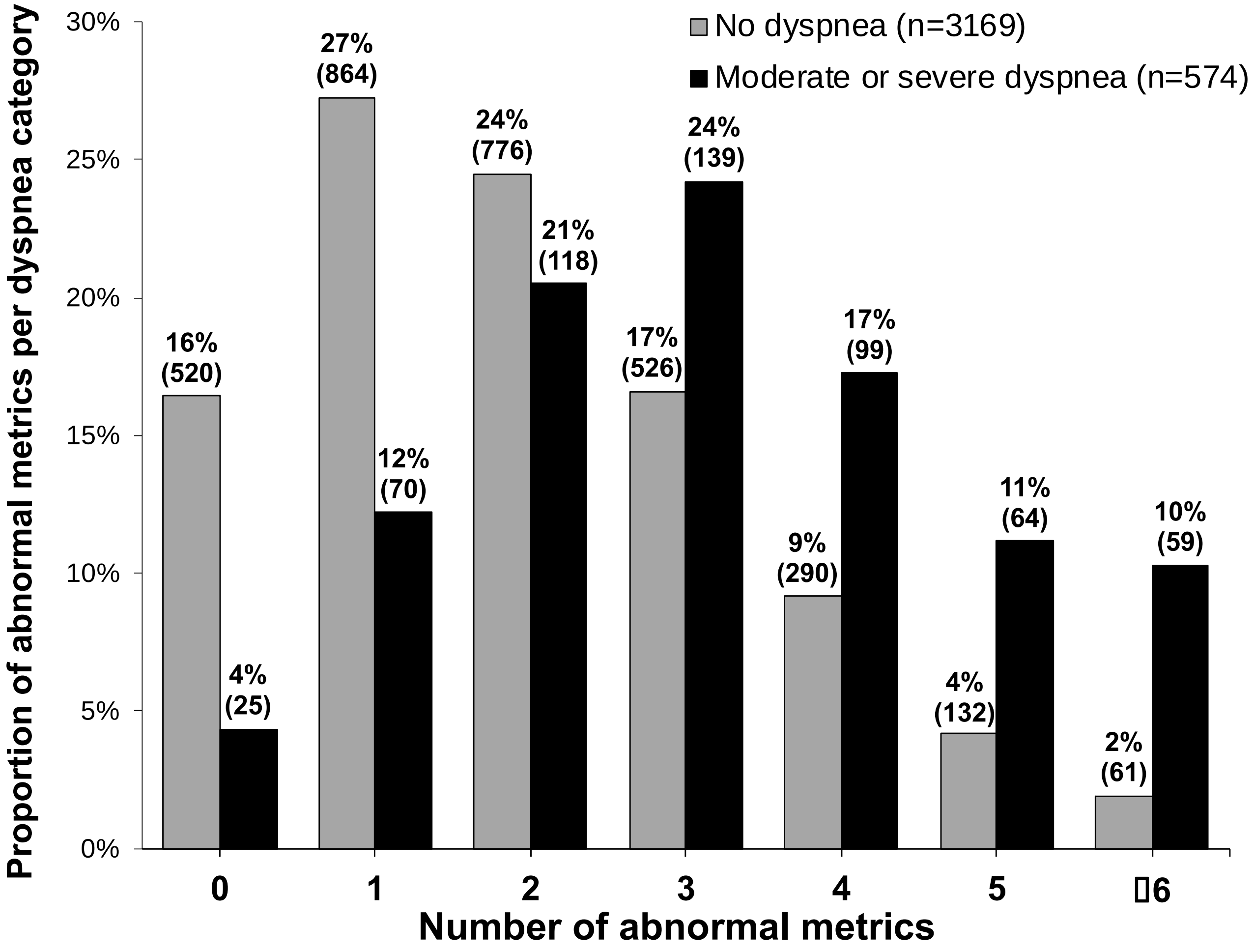

Caption: Participants with moderate/severe dyspnea had significantly more simultaneous dysfunctions (median [25-75th percentile] = 3[2-4]) than those with no/mild dyspnea (2[1-3]; p<0.001 adjusted for age, gender and race). Mild dyspnea supressed for sensitivity analysis. Participants free of heart failure, chronic obstructive pulmonary disease, body mass index ≥40 kg/m^2, or estimated glomerular filtration rate <30mL/min/1.73m^2.

eFigure 3. Adjusted Associations of Cardiovascular and Noncardiovascular Organ Dysfunction With Moderate to Severe Compared With No Dyspnea

DYSFUNCTIONS	DYSPNEA None	DYSPNEA Moderate/ Severe	POPULATION ATTRIBUTABLE RISK %
LV Hypertrophy	21%	30%	-
LV Systolic Dysfunction	25%	30%	-
LV Diastolic Dysfunction	23%	34%	-
Pulmonary Hypertension	9%	16%	3.9% (0.3-7.3)
Obstructive ventilatory pattern	29%	38%	12.9% (7.0-18.6)
Restrictive ventilatory pattern	12%	26%	12.3% (8.1-16.3)
Renal Dysfunction	23%	32%	-
Anemia	14%	27%	9.2% (4.7-13.6)
Lower limb weakness	7%	25%	7.7% (4.0-11.3)
Upper limb weakness	21%	35%	6.1% (0.4-11.5)
Depression Symptoms	3%	11%	5.0% (2.7-7.2)
Obesity	26%	45%	24.0% (17.6-29.8)

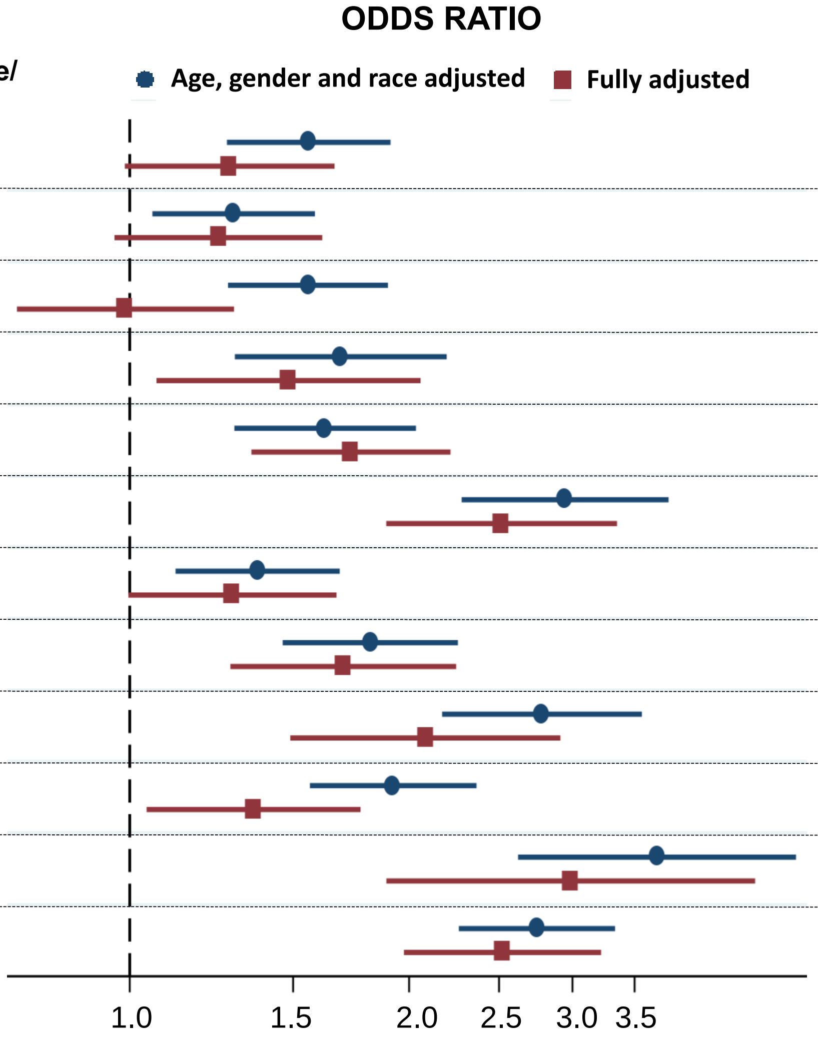

Caption: Percentage values in columns 2 and 3 represent the prevalence (%) of cardiovascular and non-cardiovascular dysfunctions in each dyspnea severity group. “Fully adjusted model”: adjusted for age, sex, race and all listed metrics of organ dysfunction. Population attributable risk is shown as a percent with 95% confidence intervals. Mild dyspnea supressed for sensitivity analysis. Participants free of heart failure, chronic obstructive pulmonary disease, body mass index ≥40 kg/m^2, or estimated glomerular filtration rate <30mL/min/1.73m^2.

© 2019 Ramalho SHR et al. *JAMA Network Open.*

eAppendix. Methods for Inverse-Probability-of-Attrition Weighting Analysis

To examine the impact of inclusion bias due to potentially non-random Visit 5 non-attendance on the studied associations, we applied inverse-probability-of-attrition weighting (IPAW). Visit 5 nonattendance was modeled among participants alive at the initiation of visit 5, without previously diagnosed HF, COPD, BMI ≥40kg/m2 and eGFR<30 mL/min/1.73m2, using the following covariates from Visit 1: age, gender, race, hypertension, diabetes, smoking status, systolic blood pressure, heart rate, body mass index and eGFR. The resulting calculated weights were incorporated into multivariable models for the association of moderate or severe dyspnea with cardiovascular and non-cardiovascular organ function. Following Tables S2 to S5 show IPAW analysis.

eTable 1. Clinical Characteristics by Dyspnea Severity Using Inverse-Probability-of-Attrition Weighting

Clinical Characteristics	Overall	No/mild	Moderate or severe	p[a]
Age (years), median [IQR]	76.6 [4.8]	75.9 [8.2]	77.9 [9.5]	<0.001
Female	60.1%	56.9%	62.4%	0.002
Black	25.1%	21.0%	33.6%	<0.001
Hypertension	84.3%	82.0%	91.2%	<0.001
Diabetes	37.0%	33.7%	49.4%	<0.001
Coronary heart disease	13.5%	12.3%	18.0%	<0.001
Stroke	3.6%	2.9%	5.1%	0.046
Current smoker	6.1%	5.5%	8.4%	0.001
Former smoker	52.1%	51.6%	53.1%	0.160

[a]p values for the comparison between dyspnea groups, adjusted for age, race and gender. Participants free of heart failure, chronic obstructive pulmonary disease, body mass index ≥40 kg/m^2, or estimated glomerular filtration rate less than 30mL/min/1.73m^2.

eTable 2. Measures of Cardiovascular Function By Dyspnea Severity Using Inverse-Probability-of-Attrition Weighting

Function metrics	Overall	No/mild	Moderate or severe	p[a]
	median[IQR]	median[IQR]	median[IQR]	
CARDIAC				
Structure				
LVMi (g/m^2)	76 [23]	75 [23]	80 [26]	<0.001
LVEDV index (mL/m^2)	42.2 [13.1]	42.4 [13.1]	41.5 [13.0]	0.928
MWT (cm)	0.96 [0.17]	0.95 [0.17]	1.00 [0.18]	<0.001
RWT (cm)	0.42 [0.08]	0.42 [0.08]	0.39[0.04]	<0.001
LV Systolic Function				
LVEF (%)	65.8 [7.3]	65.8 [7.2]	65.6 [8.0]	0.060
LS (%)	-18.1[3.2]	-18.2 [3.2]	-17.7 [3.6]	<0.001
CS (%)	-28.3 [4.9]	-28.3 [4.7]	-28.0 [5.4]	0.003
LV Diastolic Function				
LAd (cm)	3.52 [0.71]	3.49 [0.68]	3.59 [0.75]	<0.001
LAVi (mL/m^2)	24.9 [9.8]	24.7 [9.6]	25.4 [10.6]	0.006
Lateral e' (cm/s)	6.7 [2.6]	6.8 [2.6]	6.4 [2.8]	0.036
Septal e' (cm/s)	5.4 [1.8]	5.5 [1.8]	5.2 [1.8]	0.004
E/e' lateral	9.6 [4.6]	9.4 [4.4]	10.3 [5.2]	<0.001
E/e' septal	11.7 [4.9]	11.5 [4.5]	12.7 [5.8]	<0.001
Right Ventricle and Pulmonary Hemodynamic				
RVFAC	0.53 [0.11]	0.53 [0.11]	0.52 [0.12]	0.004
TAPSMV (cm/s)	11.4 [3.6]	11.4 [3.5]	11.2 [3.8]	0.569
Est PASP (mmHg)	27 [7]	27 [7]	29 [9]	<0.001
SYSTEMIC ARTERIAL				
MAP (mmHg)	87 [15]	87 [15]	88 [15]	0.127
PP (mmHg)	63 [19]	62 [19]	65 [19]	0.451
cfPWV (cm/s)	1142 [399]	1131 [383]	1209 [366]	0.092

Legend: LV: left ventricle; LVMi: LV mass index; LVEDV: LV end-diastolic volume; MWT: LV mean wall thickness; RWT: Relative wall thickness; LVEF: LV ejection fraction; LS: longitudinal strain; CS: circumferential strain; LAd: left atrium maximal anteroposterior diameter; LAVi: left atrial volume index; Lateral e': Lateral early diastolic myocardial velocity; Septal e': Septal early diastolic myocardial velocity; E/e': early mitral inflow velocity / early diastolic velocity; RVFAC: right ventricle fractional area change; TAPSVM: Tricuspid annular peak systolic myocardial velocity; Est PASP: estimated pulmonary artery systolic pressure; MAP: mean arterial pressure; PP pulse pressure; cfPWV: carotid-femoral pulse wave velocity.

[a]p values for the comparison between dyspnea groups, adjusted for age, race and gender. Participants free of heart failure, chronic obstructive pulmonary disease, body mass index ≥40 kg/m^2, or estimated glomerular filtration rate less than 30mL/min/1.73m^2.

eTable 3. Measures of Noncardiovascular Function by Dyspnea Severity Using Inverse-Probability-of-Attrition Weighting

Function metrics	Overall	No/mild	Moderate or severe	p[a]
	median [IQR]	median [IQR]	median [IQR]	
PULMONARY				
%predFEV1	95.0 [27.1]	96.5 [24.8]	86.3 [30.0]	<0.001
%pred FVC	96.4 [23.8]	97.5 [22.8]	90.2 [36.0]	<0.001
FEV1/FVC (%)	73.2 [10.3]	73.5 [9.8]	72.1 [12.8]	<0.001
RENAL				
eGFR ($mL/min/1.73m^2$)	70.3 [24.6]	71.3 [23.5]	67.1 [28.3]	0.003
HEMATOLOGIC				
Hemoglobin(g/dL)	13.3 [1.8]	13.4 [1.8]	12.0 [1.1]	0.003
PHYSICAL FUNCTION				
SPPB score	10 [3]	10 [2]	8 [4]	<0.001
Grip strength (kg)	28 [14]	28 [14]	26 [12]	<0.001
DEPRESSION				
CES-D 11 items	2 [3]	2 [3]	4 [4]	<0.001
BODY MASS				
BMI (kg/m^2)	27.7 [6.3]	27.3 [5.9]	29.5 [7.8]	<0.001

Legend: % pred: percent predicted; FEV1: forced expiratory volume in 1 second; FVC: forced vital capacity; eGFR: estimated glomerular filtration rate, using CKD-EPI equation; SPPB: Short Physical Performance Battery; CES-D 11-items: Center for Epidemiologic Studies Depression Scale – 11 items; BMI: body mass index.

[a]p values for the comparison between dyspnea groups, adjusted for age, race and gender. Participants free of heart failure, chronic obstructive pulmonary disease, body mass index ≥40 kg/m^2, or estimated glomerular filtration rate less than $30mL/min/1.73m^2$.

eTable 4. Prevalence of Cardiovascular And Noncardiovascular Dysfunction By Dyspnea Severity Using Inverse-Probability-of-Attrition Weighting

Dysfunction	Overall	No/mild	Moderate or severe	Model 1 p	Model 2 p
LV Hypertrophy	26.0%	23.7%	34.4%	<0.001	0.046
LV Systolic dysfunction	29.1%	27.1%	37.6%	<0.001	0.010
LV Diastolic Dysfunction	29.4%	26.0%	35.8%	<0.001	0.917
Pulmonary Hypertension	12.8%	10.1%	18.1%	<0.001	0.003
Obstructive pattern	38.6%	35.9%	50.7%	<0.001	<0.001
Restrictive pattern	17.7%	14.7%	30.8%	<0.001	<0.001
Renal dysfunction	29.2%	26.5%	35.4%	<0.001	0.060
Anemia	12.9%	17.7%	30.1%	<0.001	0.010
Lower Limb weakness	8.1%	10.2%	29.1%	<0.001	<0.001
Upper Limb weakness	18.9%	17.7%	23.7%	0.001	0.696
Depression	5.8%	3.8%	13.7%	<0.001	<0.001
Obesity	31.4%	28.0%	46.2%	<0.001	<0.001

Legend: LV: left ventricle; LV Hypertrophy: LV mass index (LVMi) >96.1g/m^2 men and >83g/m^2 women. Systolic dysfunction: defined as any of: LVEF<60% men and <59% women, or longitudinal strain <-16% both genders, or circumferential strain <23% for both genders. Diastolic dysfunction: defined as at least two of: left atrial (LA) anteroposterior diameter (LAD) >4.0cm men and >3.7cm women, or LA volume index (LAVi) >31mL/m^2 men and >30mL/m^2 women, or Lateral tissue Doppler imaging (TDI) relaxation velocity (e') <5.4cm/s men and <5.1cm/s women, or septal TDI e'<4.6cm/s men and <4.5cm/s women, or lateral E/e'>11.5 men and >13.3 women; or septal E/e'>13.3 men and >15.1 women. Pulmonary hypertension: pulmonary artery systolic pressure >32mmHg. Obstructive ventilatory pattern: FEV1/FVC<70%; Restrictive pattern: percent predicted forced vital capacity <80%; Renal Dysfunction: estimated glomerular filtration rate <60mL/min/1.73m^2; Anemia: hemoglobin <13g/dL men and <12g/dL women; Lower limbs physical dysfunction: short physical performance battery score≤6; Upper limb physical dysfunction: grip strength <20th percentile for gender and body mass index (BMI) predicted; Depression: CES-D 11items score ≥9 points; Obesity: BMI≥30kg/m^2.
Model1: model adjustment included age, gender and race.
Model 2: model adjustment included age, gender, race, left ventricle hypertrophy, LV Systolic dysfunction, LV Diastolic dysfunction, pulmonary artery hypertension, obstructive ventilatory pattern, restrictive ventilator pattern, renal dysfunction, anemia, lower limbs physical dysfunction, upper limb physical dysfunction, depression and obesity.

eTable 5. Clinical Characteristics and Metrics of Cardiovascular and Noncardiovascular Function by Severity of Reported Dyspnea, Adjusted for Age, Sex, Race, Household Income, Study Center, And Leisure-Time Physical Activity

Clinical characteristics	No dyspnea (mMRC=0) n=3169 (73%)	Mild dyspnea (mMRC=1) n=599 (14%)	Moderate or severe dyspnea (mMRC≥2) n=574 (13%)	p-trend[a]
**DEMOGRAPHICS**				
Age, years; mean±SD	75.4 ± 4.9	76.4 ± 5.0	77.4 ± 5.3	<0.001
Female; n(%)	1764 (56%)	397 (66%)	372 (65%)	<0.001
Black race; n(%)	563 (18%)	116 (19%)	169 (29%)	<0.001
Study Center				<0.001
Forsyth County, NC	761 (24%)	134 (22%)	84 (15%)	
Jackson, MS	511 (16%)	106 (18%)	151 (26%)	
Minneapolis, MN	1038 (33%)	182 (30%)	175 (30%)	
Washington County, MD	859 (27%)	177 (30%)	164 (29%)	
Total family income[b]				<0.001
<$16.000	301 (10%)	81 (14%)	118 (21%)	
$16.000-34.999	709 (22%)	172 (29%)	178 (31%)	
≥$35.000	1904 (60%)	296 (49%)	229 (40%)	
Leisure-time Physical activity[c]				<0.001
Ideal	1881 (60%)	243 (41%)	175 (31%)	
Intermediate	550 (17%)	115 (19%)	149 (26%)	
Poor	732 (23%)	240 (40%)	248 (43%)	
**CV DISEASE AND RISK FACTORS**				
Hypertension; n(%)	2459 (77%)	517 (86%)	510 (89%)	<0.001
Diabetes; n(%)	957 (30%)	224 (37%)	253 (44%)	<0.001
Current smoker; n(%)	149 (5 %)	35 (6%)	38 (7%)	0.023
Former smoker; n(%)	1473 (50%)	281 (50%)	261 (50%)	0.10
Coronary heart disease; n(%)	276 (9%)	58 (10%)	67 (12%)	0.003
Stroke; n(%)	59 (2%)	19 (3%)	26 (4%)	0.004
**MARKERS OF HF**				
Lower extremity edema; n(%)	345 (11%)	96 (16%)	122 (22%)	<0.001
Diuretic use; n(%)	87 (3%)	38 (6%)	45 (8%)	<0.001
NT-proBNP, pg/mL; median[25-75th percentile]	108 [58-208]	136 [71-256]	160 [81-318]	<0.001
High sensit troponin-T, ng/L; median[25-75th percentile]	10.0 [7.0-14.0]	10.0 [7.0-15.0]	12.0 [9.0-18.0]	<0.001
**CARDIAC**				
Left Ventricle Structure				
LV mass index (g/m^2); mean±SD	77 ± 18	79 ± 18	80 ± 20	<0.001
LVEDV index (mL/m^2); mean±SD	43.9 ± 10.2	42.1 ± 9.3	42.4 ± 9.3	0.15
Mean wall thickness (cm); mean±SD	0.97 ± 0.13	0.99 ± 0.13	1.01 ± 0.14	<0.001
Relative wall thickness (cm); mean±SD	0.42 ± 0.07	0.43 ± 0.08	0.44 ± 0.09	<0.001

Left ventricle Systolic Function				
LV ejection fraction (%); mean±SD	65.8 ± 5.8	65.8 ± 6.4	65.6 ± 6.4	0.44
Longitudinal strain (%); mean±SD	-18.2 ± 2.4	-18.0 ± 2.5	-17.8 ± 2.6	0.004
Circumferential strain (%); mean±SD	-28.0 ± 3.7	-27.9 ± 4.1	-27.5 ± 3.6	0.011
Left Ventricle Diastolic Function				
LA diameter (cm); mean±SD	3.49 ± 0.49	3.52 ± 0.45	3.56 ± 0.54	<0.001
LA volume index (mL/m^2); mean±SD	25.1 ± 8.3	26.2 ± 7.8	26.4 ± 8.8	<0.001
Lateral e' (cm/s); mean±SD	7.2 ± 2.0	6.9 ± 1.8	6.8 ± 2.1	0.15
Septal e' (cm/s); mean±SD	5.8 ± 1.5	5.5 ± 1.3	5.5 ± 1.6	0.004
E/e' lateral; mean±SD	9.8 ± 3.6	10.3 ± 3.5	10.6 ± 4.0	0.001
E/e' septal; mean±SD	11.8 ± 3.9	12.6 ± 3.8	13.0 ± 4.7	<0.001
Right Ventricle (RV) function and pulmonary hemodynamics				
RV fractional area change; mean±SD	0.52 ± 0.08	0.53 ± 0.8	0.52 ± 0.08	0.52
Tricuspid annular peak systolic myocardial velocity (cm/s); mean±SD	11.9 ± 2.8	11.7 ± 3.0	11.7 ± 3.0	0.42
Estimated PASP (mmHg); mean±SD	27 ± 5	29 ± 7	29 ± 6	<0.001
**SYSTEMIC ARTERIAL**				
Systolic pressure (mmHg); mean±SD	130 ± 17	131 ± 19	131 ±18	0.51
Diastolic pressure (mmHg); mean±SD	67 ± 10	67 ± 11	66 ± 11	0.82
Pulse pressure (mmHg); mean±SD	63 ± 14	64 ± 15	65 ± 14	0.57
cfPWV (cm/s); mean±SD	1154 ± 337	1170 ± 293	1213 ± 348	0.71
**PULMONARY**				
% predicted FEV_1; mean±SD	98.3 ± 18.5	91.9 ± 19.1	89.4 ± 22.4	<0.001
% predicted FVC; mean±SD	99 ± 19	94 ± 18	91 ± 22	<0.001
FEV_1/FVC (%); mean±SD	73.2 ± 7.4	72.9 ± 8.4	71.3 ± 10.0	<0.001
**RENAL**				
eGFR (mL/min/1.73m^2) ; mean±SD	71.9 ± 15	69.8 ± 16	68.5 ± 17	0.002
**HEMATOLOGIC**				
Hemoglobin (g/dL); mean±SD	13.5 ± 1.3	13.3 ± 1.4	12.9 ± 1.5	<0.001
**PHYSICAL FUNCTION**				
SPPB score; mean±SD	9.9 ± 2.1	9.4 ± 2.2	8.1 ± 2.8	<0.001
Grip strength (kg); mean±SD	30.2 ± 10.5	27.7 ± 9.7	26.9 ± 9.4	<0.001
**DEPRESSION SCORE**				
CES-D 11-items; mean±SD	2.4 ± 2.5	3.5 ± 2.9	4.1 ± 3.3	<0.001
**BODY MASS**				
Body mass index (kg/m^2); mean±SD	27.3 ± 4.3	29.2 ± 4.5	29.6 ± 4.9	<0.001

Legend: CV: cardiovascular. Hypertension: use of antihypertensive medication or blood pressure ≥140/90mmHg. Diabetes: fasting glycemia ≥126 mg/dL, nonfasting glycemia ≥200 mg/dL or use of anti-diabetic medication. LV: left ventricle; LVEDV: LV end-diastolic volume; LA: left atrium; Lateral e': Lateral early diastolic myocardial velocity; Septal e': Septal early diastolic myocardial velocity; E/e': early mitral inflow velocity/early diastolic velocity; PASP: pulmonary artery systolic pressure; cfPWV: carotid-femoral pulse wave velocity; FEV1: forced expiratory volume in 1 second; FVC: forced vital capacity; eGFR: estimated glomerular filtration rate, using CKD-

EPI equation; SPPB: Short Physical Performance Battery; CES-D 11-items: Center for Epidemiologic Studies Depression Scale – 11 items.
[a]Adjusted p values for age, race and gender to all variables, except for Age, Female, Black race, Study center, Income and Physical activity. Participants were free of diagnosed HF or COPD, BMI≥40 kg/m2, or eGFR<30mL/min/1.73m2.
[b] 8% missing
[c] Leisure-time physical activity: Ideal ≥150 min/week of moderate activity, ≥75 min/week of vigorous activity, or ≥150 min/week of moderate + vigorous activity; Intermediate 1–149 min/week of moderate activity, 1–74 min/week of vigorous activity, or 1–149 min/week of moderate + vigorous activity; Poor 0 min/week of moderate + vigorous activity.

eTable 6. Associations of Cardiovascular and Noncardiovascular Organ Dysfunctions With Moderate to Severe Compared With No to Mild Dyspnea Adjusted For Socioeconomic Characteristics

**Dysfunctions**	**Model 1: adjusted for age, gender, race, study center, household income and physical activity[a]**	**Model 2: adjusted as model 1 and all listed dysfunctions**
	OR (95%CI)	OR (95%CI)
LV Hypertrophy	1.40 (1.14-1.71)	1.22 (0.95-1.58)
LV Systolic Dysfunction	1.21 (0.99-1.48)	1.20 (0.94-1.58)
LV Diastolic Dysfunction	1.45 (1.19-1.76)	0.99 (0.76-1.28)
Pulmonary hypertension	1.51 (1.16-1.96)	1.45 (1.06-1.98)
Obstructive pattern	1.65 (1.32-2.07)	1.72 (1.35-2.19)
Restrictive pattern	2.43 (1.89-2.12)	2.06 (1.57-2.71)
Renal Dysfunction	1.26 (1.03-1.54)	1.26 (0.98-1.61)
Anemia	1.64 (1.32-2.03)	1.55 (1.18-2.03)
Lower limbs weakness	2.40 (1.88-3.07)	1.96 (1.42-2.69)
Upper limbs weakness	1.71 (1.39-2.11)	1.26 (0.97-1.61)
Depression symptoms	2.61 (1.88-3.63)	2.11 (1.38-3.23)
Obesity	2.04 (1.69-2.47)	2.02 (1.59-2.57)

[a]Study center: Forsyth County NC, Jackson MS, Minneapolis MN, Washington County MD. Household Income: Lower (<USD 16,000), Intermediate (USD 16,000-34,999), Higher (≥35,000); Leisure-time physical activity: Ideal ≥150 min/week of moderate activity, ≥75 min/week of vigorous activity, or ≥150 min/week of moderate + vigorous activity; Intermediate 1–149 min/week of moderate activity, 1–74 min/week of vigorous activity, or 1–149 min/week of moderate + vigorous activity; Poor 0 min/week of moderate + vigorous activity. Participants free of heart failure, chronic obstructive pulmonary disease, body mass index ≥40 kg/m^2, or estimated glomerular filtration rate <30mL/min/1.73m^2.

© 2019 Ramalho SHR et al. *JAMA Network Open.*
